# Optimizing surfactant removal from a soft-templated ordered mesoporous carbon precursor: an *in situ* SAXS study

**DOI:** 10.1107/S1600576723003886

**Published:** 2023-05-29

**Authors:** Max Valentin Rauscher, Malina Seyffertitz, Richard Kohns, Sebastian Stock, Heinz Amenitsch, Nicola Huesing, Oskar Paris

**Affiliations:** a Chair of Physics, Department Physics, Mechanics and Electrical Engineering, Montanuniversität Leoben, Leoben, Austria; b Department of Chemistry and Physics of Materials, Paris Lodron University Salzburg, Salzburg, Austria; c Institute of Inorganic Chemistry, Graz University of Technology, Graz, Austria; Brazilian Synchrotron Light Laboratory, Brazil

**Keywords:** *in situ* small-angle X-ray scattering, soft-templated carbons, ordered mesoporous carbons, calcination

## Abstract

*In situ* small-angle X-ray scattering (SAXS) was employed to identify critical parameters during thermal treatment for template removal of an ordered mesoporous carbon precursor synthesized via a direct soft-templating route. The structural parameters obtained from the SAXS data as a function of time were the lattice parameter of the 2D hexagonal structure, the diameter of the cylindrical mesostructures and a power-law exponent characterizing the interface roughness. Moreover, detailed information on contrast changes and pore lattice order was obtained from analysis of the integrated SAXS intensity of the Bragg and diffuse scattering separately. Five characteristic regions during heat treatment were identified and discussed regarding the underlying dominant processes. The influence of temperature and O_2_/N_2_ ratio on the final structure was analyzed, and parameter ranges were identified for an optimized template removal without strongly affecting the matrix. The results indicate that the final structure and controllability of the process are optimum for temperatures between 260 and 300°C with a gas flow containing 2 mol% of O_2_.

## Abstract 2

*In situ* small-angle X-ray scattering was used to identify structural changes during thermal treatment of an ordered mesoporous carbon precursor material, synthesized via direct soft templating, to remove Pluronic F127 surfactant from a resorcinol–formaldehyde frame.

## 1. Introduction

Ordered mesoporous carbons (OMCs) have attracted significant attention in recent years due to their unique characteristics, which include chemical and thermal stability, electrical conductivity, high surface area, and large pore volume (Ndamanisha & Guo, 2012 ▸). Other key benefits of OMCs are a tunable pore size (distribution) in the mesopore regime, as well as relatively low production costs and easy handling (Sakintuna & Yürüm, 2005 ▸). The possibility of introducing microporosity in the mesopore walls of OMCs via physical or chemical activation renders these hierarchically structured micro/meso-porous materials highly attractive for a wide range of applications, such as molecular sieves (Zhou *et al.*, 2005 ▸), separation of CO_2_/CH_4_ mixtures (Yuan *et al.*, 2013 ▸), adsorption of environmental pollutants (Gang *et al.*, 2021 ▸), catalyst support (Liu *et al.*, 2008 ▸), hydrogen storage (Jeong *et al.*, 2021 ▸) and electrochemical sensing (Ndamanisha & Guo, 2012 ▸), as well as high-power electrode material for supercapacitors (Koczwara *et al.*, 2019 ▸).

Various approaches for the synthesis of OMCs have been proposed since their independent introduction by Ryoo *et al.* (1999 ▸) and Hyeon’s group (Lee *et al.*, 1999 ▸). Today, nanocasting and direct soft templating have prevailed as the two main strategies. Nanocasting involves the preparation of an ordered mesoporous template, typically silica, and the impregnation of its mesopores with a carbon precursor material. The precursor is subsequently carbonized and the template is removed using acid-aided etching. This results in OMCs composed of the negative replication of the mesoporous template (Lu & Schüth, 2006 ▸). As nanocasting involves complex and intricate synthesis steps, and requires a mesoporous mold which is removed upon etching, it is often regarded as costly, unresourceful and time consuming and, thus, hardly scalable for industrial production. Consequently, direct soft-templating approaches, which do not require negative pore space filling of a template, have gained increasing popularity for the production of OMCs. This synthesis strategy is based on the self-assembly of block-copolymer surfactants in the presence of phenolic resin into ordered mesostructures of a specific symmetry, such as 2D hexagonal (*P*6*mm*), 3D bicontinuous ( 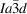 ), body-centered cubic ( 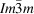 ) and *L*α lamellar (Meng *et al.*, 2006 ▸). During calcination, surfactant micelles are thermally decomposed giving way to the mesopore space. The remaining phenolic resin frame is then carbonized to yield the final OMC structure (Ma *et al.*, 2013 ▸). However, although this synthesis strategy requires less intricate steps compared with nanocasting, soft templating still holds some major challenges.

A crucial factor is the mesoporous structure, which is only attainable through calcination at a temperature where the polymeric resin remains thermally stable. The temperature window for this process is quite small, and therefore, it proves difficult to narrow down a reliable parameter space where surfactants are completely removed but the intended ordered structure is fully maintained. For a common surfactant/resin pairing, Pluronic F127 PEO–PPO–PEO triblock copolymer and resorcinol–formaldehyde (RF) resin gel, calcination was reported in a timeframe between 10 and 60 min at temperatures of 250 to 350°C in nitro­gen atmosphere with the addition of 2 to 10 mol% of oxygen (Putz *et al.*, 2020 ▸; Hasegawa *et al.*, 2016 ▸; Robertson *et al.*, 2022 ▸; Ma *et al.*, 2013 ▸). So far, a systematic parameter study to determine the mechanisms at play for different temperatures, holding times and oxidizing atmospheres, and their influence on surfactant removal, is still missing. This could be due to the inherent difficulty of *in situ* monitoring of such complex processes like the thermal degradation of a triblock copolymer within the confinement of a phenolic resin frame over several length scales in a sufficiently large sample volume. Small-angle scattering is a well established technique for probing (ordered) nanostructured materials and has been successfully employed to investigate structurally similar ordered mesoporous silicas during synthesis (Flodström *et al.*, 2004 ▸; Khodakov *et al.*, 2005 ▸), after shear-induced anisotropy (Putz *et al.*, 2017 ▸), during pore filling and emptying (Erko *et al.*, 2010 ▸), during physisorption of films and capillary condensation (Zickler *et al.*, 2006 ▸), and during water-adsorption-induced deformation (Morak *et al.*, 2017 ▸). In this work, we present a time-resolved *in situ* small-angle X-ray scattering (SAXS) investigation of the calcination step of an RF resin/Pluronic F127 mesostructure. The *in situ* SAXS data not only help to elucidate the effect of calcination under the influence of temperature, isothermal holding time and oxygen content on the resulting structure on the nanometre scale but also allow one to propose a safe parameter range for successful calcination.

## 2. Experimental

### 2.1. Chemicals

Pluronic F127 PEO–PPO–PEO (EO_106_PO_70_EO_106_) triblock copolymer, resorcinol (99%) and tri­ethyl­ene glycol (TEG, 99%) were purchased from Sigma–Aldrich (St Louis, Missouri). Formaldehyde solution (37 wt%, stabilized with methanol) and 1,3,5-tri­methyl­benzene (TMB, >98%) were provided by Merck (Darmstadt, Germany). Benzyl alcohol (BnOH, analytical grade), hydro­chloric acid (HCl, 32%) and 2-propanol (i-PrOH, technical grade) were purchased from VWR (Darmstadt, Germany). All chemicals were used as received, without further purification. Aqueous solutions were prepared using deionized water with a specific resistance of 15 MΩ cm.

### 2.2. Synthesis of the OMC precursor

**Figure 1:**
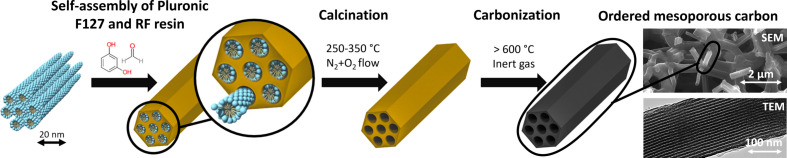
Schematic direct soft synthesis route of an OMC using Pluronic F127 and RF resin. The rightmost part shows scanning electron microscopy (SEM) (upper part) and transmission electron microscopy (TEM) images (lower part) of a monolithic carbonized sample, visualizing the framework of struts and the ordered mesopores within one strut.

The synthesis of the direct soft-templated precursor monoliths was adapted according to the procedure described by Hasegawa *et al.* (2016 ▸) and is schematically depicted in Fig. 1 ▸.

Gel preparation was conducted by adding, in successive steps, 25 ml of 1*M* HCl, 15 ml of TMB, 15 ml of BnOH, 15 g of F127 and 11 g of resorcinol to 150 ml of TEG under vigorous stirring. After the solution became homogeneous, 15 ml of formaldehyde solution was added with additional stirring for 30 min and subsequent sealing in plastic containers. The solution was then allowed to gel and age at 60°C for 48 h. After gelation, the gels were washed in i-PrOH which was refreshed several times over five days to extract the residual moieties. Thereafter, the gel was supercritically dried in an autoclave with CO_2_ (*T*_c_ = 31°C; *P*_c_ = 7.38 MPa) at 60°C and 10 MPa with a slow depressurization for at least 5 h, as described in more detail by Kohns *et al.* (2023 ▸). This resulted in a disc-shaped OMC precursor monolith.

### 2.3. *In situ* SAXS during calcination

*In situ* SAXS measurements were conducted at the Austrian SAXS beamline at ELETTRA Sincrotrone Trieste, Italy (Amenitsch *et al.*, 1998 ▸). A custom-built setup consisting of a heating unit and a quartz capillary sample holder with a gas-line connection in a flow-through configuration was used for *in situ* calcination. Cylindrical samples with a diameter of 1.5 mm and a length of ∼5 mm were punched out of the monolith and degassed in a vacuum at room temperature for at least 24 h prior to the measurements. A new sample was placed into the capillary for each measurement run and kept in place using a type N thermocouple inserted at the opposing end of the capillary. This ensured precise temperature monitoring and a stable axial position of the sample within the beam. Each measurement run consisted of a heating phase, in which the sample temperature was increased at a rate of 3 or 10°C min^−1^, and an isothermal holding phase at temperatures between 260 and 340°C. The heating was performed in a pure N_2_ atmosphere, with the addition of 2 or 10 mol% of O_2_ during the isothermal holding.

The atmosphere in the capillary was regulated using calibrated mass-flow controllers (Sierra Instruments, Monterey, California) for the used gases, *i.e.* nitro­gen and dried synthetic air. During heating and isothermal holding, scattering signals were recorded using a 2D Pilatus3 1M detector (Dectris Ltd, Baden-Daettwil, Switzerland) at a detector distance of 760 mm with an acquisition time of 10 s, followed by 10 s of waiting time. The beam size was 0.3 mm × 2 mm (v × h) resulting in an irradiated sample volume of ∼0.8 mm^3^. The used photon energy was 8 keV (wavelength λ = 0.154 nm), which allowed us to cover a *q* range between 0.2 and 10 nm^−1^ [ 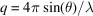 with 2θ being the scattering angle]. Two-dimensional scattering patterns were subjected to standard normalization and correction procedures at the beamline (primary intensity, transmission, exposure time), and subsequently azimuthally integrated (Burian *et al.*, 2022 ▸).

### 2.4. SAXS data treatment and fitting procedure

**Figure 2:**
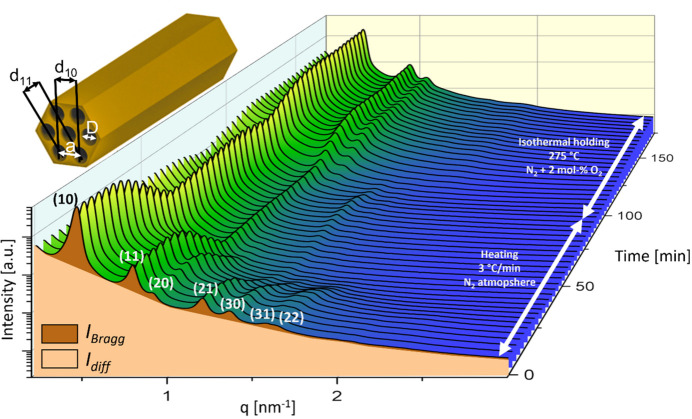
*In situ* SAXS profiles for heating in N_2_ atmosphere followed by calcination at 275°C using 2 mol% of O_2_. The highlighted areas underneath the first profile correspond to the diffuse intensity *I*_diff_ (light color) and the Bragg intensity *I*_Bragg_ (dark color). Bragg peaks originating from the 2D hexagonal structure, as indicated in the inset in the top left, are indexed with 2D Miller indices.

For SAXS data analysis, a single-step model consisting of two homogeneous phases, involving a matrix phase and the (initially surfactant filled) mesopore space, was employed for the assessment of model-based parameters. A core–shell cylinder model was also considered to describe the processes during calcination more accurately, but was practically not feasible due to the low scattering contrast of PEO and PPO with respect to RF after water removal. Following earlier work on mesoporous silica (Findenegg *et al.*, 2010 ▸), the scattering intensity was separated into two contributions. The presence of sharp Bragg peaks in the scattering profile, as indicated in Fig. 2, can be attributed to the presence of a 2D hexagonal structure. This intensity contribution is referred to as the Bragg intensity (*I*_Bragg_) and can be described as the product of the structure factor [*S*(*q*)], the form factor [|*F*(*q*)|^2^] and an unknown (constant) scaling factor *K*, since no absolute intensity calibration was performed. The remaining scattering intensity is referred to as the diffuse scattering intensity (*I*_diff_). Combining the approaches described by Findenegg *et al.* (2010 ▸) and Zickler *et al.* (2006 ▸), the total scattering intensity is then approximated by the incoherent sum of these two contributions:

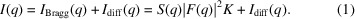

The integrated intensity 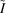 is calculated by (Glatter, 2018 ▸)

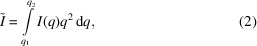

with *q*_1_ = 0.1 nm^−1^ and *q*_2_ = 7 nm^−1^, and no extrapolation in the ranges 0 < *q* < *q*_1_ and *q*_2_ < *q* < ∞ was performed. The separation of the integrated intensity into diffuse 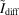 and Bragg 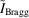 contributions was performed as described by Findenegg *et al.* (2010 ▸). The form factor [|*F*(*q*)|^2^] was approximated by a model of infinitely long monodisperse cylindrical objects (single-step model), based on the approach introduced by Impéror-Clerc *et al.* (2000 ▸). The scattering amplitude *F*(*q*) of the cylinder cross section is given by

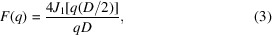

where 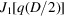 is the Bessel function of the first kind and first order, and *D* is the diameter of the cylinder. The spherical average of the structure factor for a perfect 2D hexagonal arrangement of long cylinders is given by (Zickler *et al.*, 2006 ▸)

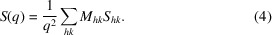

Here, the multiplicity factor *M_hk_* for the diffraction peaks is 12 for mixed Miller indices and 6 for Miller indices described by (*h*0) and (*hh*). Furthermore, *S_hk_* are delta functions at positions *q_hk_*. The lattice parameter *a* describes the distance between the centers of two neighboring cylinders:

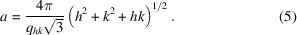

Bragg peaks in the scattering profiles were fitted using pseudo-Voigt functions (Newville *et al.*, 2014 ▸) and a linear spline background. The integrated intensities of each individual Bragg peak, 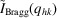 , were used to fit the form factor [|*F*(*q*)|^2^] by minimizing the following equation according to (Zickler *et al.*, 2006 ▸)

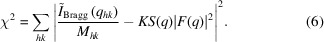

For the diffuse scattering in the high-*q* regime from 2.25–3.25 and 4.5–6.5 nm^−1^ (areas highlighted in Fig. S1 of the supporting information), the SAXS curve was fitted with a power law where the power-law exponent is not fixed at −4 (Porod’s law) but can vary between −2 and −4 for fractal surfaces (Bale & Schmidt, 1984 ▸):

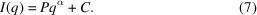

In this equation, *P* is the modified Porod constant, α is the power-law exponent describing the nature of the interface of the two constituting phases, and *C* is an additive constant related to incoherent and liquid-like scattering contributions.

## 3. Results

Fig. 2 ▸ displays the *in situ* scattering profiles of the OMC precursor material heated in N_2_ atmosphere to a final temperature of 275°C at a heating rate of 3°C min^−1^. After the final temperature had been reached, the gas atmosphere was changed to N_2_/2 mol% of O_2_ with subsequent isothermal holding at 275°C. These calcination settings are used in the following to exemplarily describe the general processes during calcination. Corresponding detailed data for other temperature and oxygen settings are shown in Figs. S2–S5.

**Figure 3:**
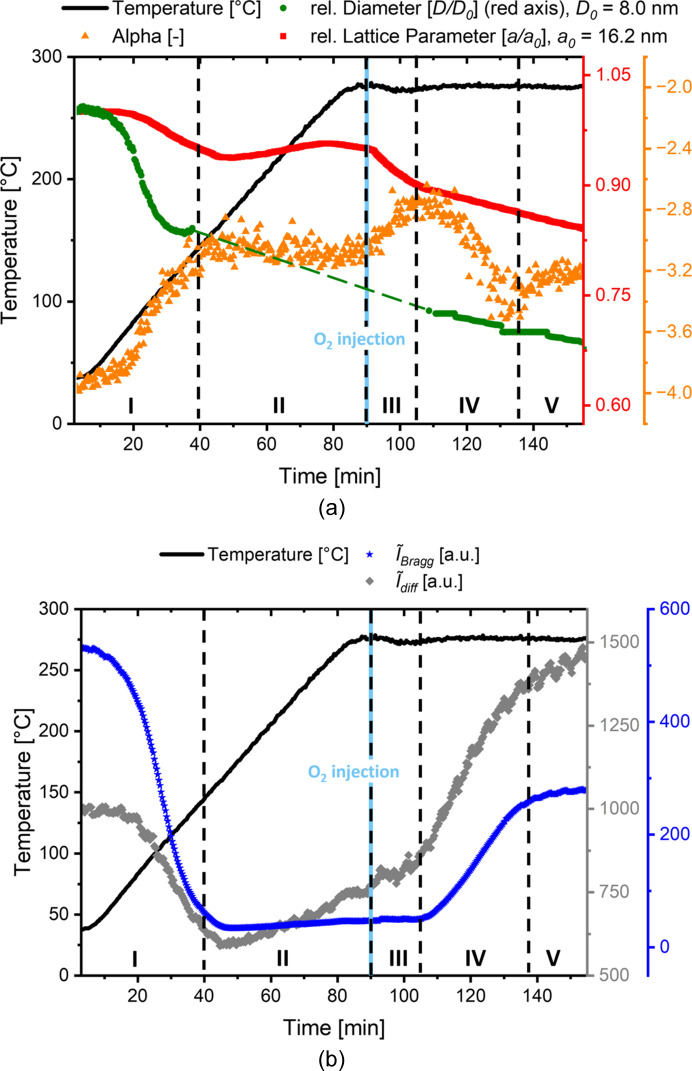
The progression of *in situ* SAXS derived (*a*) structural and (*b*) contrast parameters for heating in N_2_ atmosphere followed by calcination at 275°C in N_2_ atmosphere with the addition of 2 mol% of O_2_ (see the main text). The red scale on the right hand side of (*a*) is valid for both the relative diameter and the relative lattice parameter.

Distinct Bragg peaks at characteristically *q*-spaced positions indicate the expected 2D hexagonal structure (*P*6*mm*), which is schematically depicted in the inset of Fig. 2 ▸. From these scattering profiles, the progression of five parameters was determined as a function of time, see Figs. 3 ▸(*a*) and 3 ▸(*b*). For reference, the applied temperature profile is also added as a black line graph in Figs. 3 ▸(*a*) and 3 ▸(*b*), and the introduction of 2 mol% of O_2_ to the N_2_ gas flow, which enables the process of surfactant removal, is marked with a light blue vertical line at 90 min.

While Fig. 3 ▸(*a*) shows parameters describing structural and interfacial changes, Fig. 3 ▸(*b*) displays parameters attributed to changes in the SAXS contrast, which is related to the pore-filling state and physical and chemical changes within the sample on the nanoscale. The relative lattice parameter *a*/*a*_0_ describes the average distance between two neighboring pore centers relative to a starting lattice parameter of *a*_0_ = 16.2 nm. The relative diameter *D*/*D*_0_ describes the average diameter of the cylindrical entities relative to a starting diameter of *D*_0_ = 8.0 nm. As will be discussed in more detail further below, the pore diameter *D* could not be evaluated in the region between 40 and 105 min for this sample. In this region, a dashed line is added to Fig. 3 ▸(*a*) as a simple guide for the eye, but the actual progression of the diameter might be different. The dimensionless parameter α in Fig. 3 ▸(*a*) represents the power-law exponent of the modified Porod law [equation (7)] and is taken as a qualitative measure of the interface roughness. The closer α is to −4, the better defined (’sharper’) the interface between two phases of different electron density can be considered. A value of −4 < α < −2 indicates a ‘fractal surface’ but can also be attributed to the presence of corrugations (*e.g.* micropores) at the interface, which results in a fuzzy progression of the electron density at the interface of the two constituting phases (Rieker *et al.*, 1999 ▸, 2000 ▸; Bale & Schmidt, 1984 ▸). The integrated Bragg intensity, 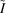 _Bragg_, which is displayed as a function of time in Fig. 3 ▸(*b*), can be attributed to two main contributions. On the one hand, 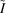 _Bragg_ is proportional to the SAXS contrast and therefore determined by the difference in the average electron density of the matrix and the (originally filled) ordered mesopore. On the other hand, the regularity (or perfection) of the mesopores also influences 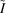 _Bragg_ and therefore renders 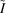 _Bragg_ a measure of the integrity of the underlying ordered mesostructure. If the deviations from perfect order were completely random, this would be described by a (static) Debye–Waller factor. More generally, lattice distortion would lead to a decrease in the Bragg integrated intensity and an increase in the diffuse integrated intensity. Therefore, deviations from ideal ordering and a decrease in scattering contrast both reduce 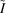 _Bragg_. The diffuse intensity, 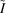 _diff_, can be mainly attributed to non-ordered scattering contributions from irregularities, such as randomly distributed micropores, defects and impurities (Findenegg *et al.*, 2010 ▸). Contributions to 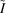 _Bragg_ and 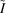 _diff_ are also shown as highlighted areas in Fig. 2 ▸.

From Figs. 3 ▸(*a*) and 3 ▸(*b*), five different stages, marking distinct changes in the progression of two or more of the evaluated parameters, were identified: stage I, from the beginning until 40 min (corresponding to a temperature of ∼150°C); stage II, from 40 to 90 min (until reaching the final temperature); stage III, immediately after the addition of O_2_ to the atmosphere at 90 min until 105 min; stage IV, from 105 until ∼137 min; and stage V, from ∼137 min onwards. In the following, we describe the changes within the different stages in some more detail, and, in the next section[Sec sec4], we provide a discussion of the possible effects taking place.

Stage I. During this stage, both 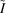 _Bragg_ and 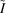 _diff_ [Fig. 3 ▸(*b*)], as well as the lattice parameter *a* and the pore diameter *D* [Fig. 3 ▸(*a*)], decrease while the exponent α is increasing. Thus, this stage can be characterized by an overall shrinking of the structure, and by a strong decrease in scattering contrast, attributed to water removal. Yet, the relative decrease of the diameter is different from the relative decrease of the lattice parameter, which means that simple homogeneous shrinkage cannot be the only effect here. This will be discussed later. The apparently sharp interface at the beginning (exponent α = −4) gradually roughens, indicated by the decrease of the exponent α. The end of stage I after ∼40 min is indicated by the clear change of the slopes of all the parameters including α.

Stage II. Between 40 and 90 min, all the parameters remain roughly constant, except for slightly increasing values of the diffuse scattering and the lattice parameter. The very low intensity and reduced number of observable Bragg peaks (see Fig. 2 ▸) make the model-based fit of the diameter *D* highly unreliable. Therefore, this parameter is omitted within stages II and III, and only a guide for the eye displaying linear change is indicated in the figure.

Stage III. The start of this stage after 90 min is determined by the addition of oxygen to the nitro­gen gas flow upon reaching the calcination temperature. A rapid decrease of the lattice parameter and an increasing exponent α are visible in Fig. 3 ▸(*a*), which indicates shrinkage of the structure and further roughening of the interface. Interestingly, the Bragg part of the integrated intensity remains constant, and also the slope of the continuously increasing diffuse part of the integrated intensity remains unchanged compared with Stage II [Fig. 3 ▸(*b*)], even though the sample has now reached a constant temperature. This intermediate stage could only be identified while heating under N_2_ at moderate temperatures between 260 and 294°C.

Stage IV. After a delay of ∼15 min (stage III), stage IV is initiated by the sudden increase of 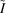 _diff_ and 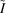 _Bragg_, as seen in Fig. 3 ▸(*b*), accompanied by a clear change in the slopes of both the lattice parameter and α. During this stage, the pore diameter *D* could again be determined with the model-based approach. Interestingly, the relative diameter *D* in this region (and also in the following stage V) experiences the same relative change compared with the lattice parameter *a*, indicating a simple shrinkage of the whole structure. The end of stage IV is determined by 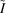 _Bragg_ reaching a clear plateau, and both 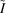 _diff_ and α changing their slopes after ∼137 min, initiating the onset of the final stage. Thus, stage IV is related to the actual calcination, *i.e.* the oxidative removal of the block-copolymer micellar structures.

Stage V. During this stage 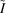 _diff_ exhibits a further slight increase, with a slope similar to stages II and III, while the slopes of both α and 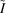 _Bragg_ reach a final plateau value. The trend of the similar decrease of lattice parameter and pore diameter is continued throughout this stage.

**Figure 4:**
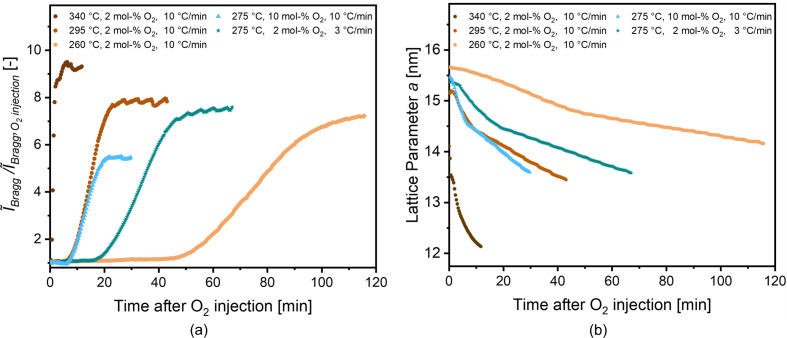
(*a*) Progression of the relative integrated Bragg intensity, 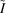 ^rel^_Bragg_, in stages III and IV, normalized to the value at the beginning of stage III ( 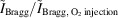 ). (*b*) Progression of the lattice parameter. For clarity, 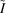 ^rel^_Bragg_ and the corresponding lattice parameter are only depicted after the addition of O_2_ to the N_2_ gas flow after the final calcination temperature has been reached.

A qualitatively similar progression of the parameters is also found for other temperatures and oxygen content (see Figs. S2–S5), and five stages can be similarly identified, except for the highest temperature studied (Fig. S2). Results for a set of five samples with varying temperature and oxygen content are shown in Fig. 4 ▸. The heating rate was not the same for all samples (3°C min^−1^ for the sample shown in Figs. 2 ▸ and 3 ▸, and 10°C min^−1^ for the other four samples), which will be discussed further below. Fig. 4 ▸(*a*) displays the relative increase of the integrated Bragg intensity within the stages III–V, *i.e.* after reaching the isothermal condition and the addition of oxygen, for different temperatures and oxygen contents. Fig. 4 ▸(*b*) shows the corresponding changes for the lattice parameter *a*. All shown samples were heated to temperatures between 260 and 340°C under N_2_ gas flow, with addition of either 2 mol% of O_2_ or 10 mol% of O_2_ after reaching the final temperature. Importantly, 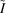 ^rel^_Bragg_ is normalized to the value at the beginning of stage III, which is different from normalizing it to the starting situation at the beginning of stage I. Since the changes in stages I and II are, however, qualitatively similar for all samples, we consider this representation more meaningful.

For the three samples subjected to a low oxygen content of 2 mol% of O_2_ at different temperatures [brown circular symbols in Fig. 4 ▸(*a*)], the final plateau value of 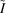 ^rel^_Bragg_ is similar for 260 and 295°C, and somewhat higher for 340°C. The time needed until the plateau value of 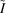 ^rel^_Bragg_ is reached, however, increases strongly with decreasing temperature. The corresponding evolution of the lattice parameters illustrates three strongly different (final) levels of contraction. Indeed, while the sample calcined at the lowest temperature experienced a decrease in the lattice parameter of ∼10% with respect to the original state (*a*_0_ = 16.2 nm), the sample calcined at the highest temperature experienced a corresponding decrease of ∼25%.

Fig. 4 ▸ also shows data from a sample calcined at a higher oxygen content of 10 mol% of O_2_ at 275°C. Compared with the sample calcined at a higher temperature (295°C) with lower oxygen content (2 mol% of O_2_), it exhibits essentially the same initial slope after oxygen injection, while reaching significantly lower values of 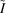 ^rel^_Bragg_. Hence, the increased reaction speed observed at higher temperatures in one sample is reached by having a higher oxygen content in the other samples at lower temperature.

## 4. Discussion

**Figure 5:**
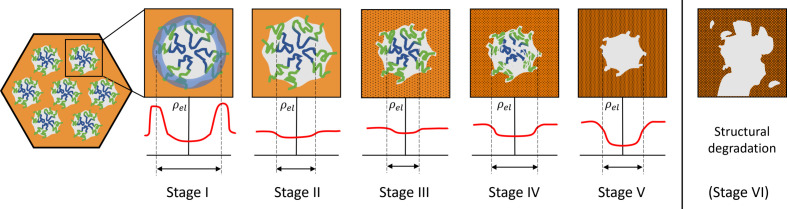
A schematic representation of the cross section of a single cylindrical mesostructure at different calcination stages, with approximated visualization of corresponding average electron-density profiles.

On the basis of the parameters derived from SAXS (see Fig. 3 ▸), we have identified five different stages of structural and compositional changes that will be discussed in more detail in this section. For a better understanding of the mechanisms at play in each stage, schematic drawings and corresponding average electron-density profiles and the perceived diameter across one mesopore are depicted in Fig. 5 ▸.

Stage I. The high contrast in combination with the ‘sharp’ interface at the very beginning is most likely attributable to a considerable amount of residual water, even after placing the monolith in a vacuum at room temperature. A water-containing corona around the folded PEO–PPO–PEO surfactant triblock copolymer is in accordance with reported water-rich regions around the outer layer of triblock-copolymer micelles (Šturcová *et al.*, 2010 ▸; Ruthstein *et al.*, 2004 ▸), and the proposed structure is depicted in Fig. 5 ▸. As the temperature is increased, the material dehydrates and the mesostructure experiences shrinkage. Hence, both the perceived pore diameter *D* and the lattice parameter *a* are expected to decrease during stage I. The exponent α is close to −4 at the beginning, indicating the sharp interface of the water-rich domain. Without estimating quantitative numbers, the scattering contrast of a water-rich region in the hydro­philic part of the micelles is expected to be considerably higher than the contrast between the dry RF matrix and the triblock copolymers. Therefore, the strong decrease of the integrated intensity during drying is comprehensible. However, the relative decrease in the lattice parameter in Fig. 3 ▸ in stage I is considerably smaller than the relative decrease of the diameter. There could be two different reasons for this observation: either a strongly different shrinkage of the block-copolymer micelles compared with the RF matrix or a different length scale dominating the scattering contrast for the hydrated and dehydrated structures. Neither scenario is describable by a single-step (*i.e.* 2-phase) model. We propose here the situation sketched in Fig. 5 ▸ for stage I, *i.e.* a relatively narrow water-rich region of higher electron density with the hydro­phobic part of the micelles and (non-hydrated part of) the RF matrix having similar (lower) electron density. Such an effective hollow-shell structure gives a very similar quality fit to the model compared with a single-step model, but appears to be more realistic. The starting diameter for an equivalent single-step model would correspond to the average value of the inner and outer diameters of the water shell. Assuming that after complete drying the contrast is determined by the electron-density difference between the hydro­phobic core on the one hand and the hydro­philic shell interdigitated with the RF matrix on the other hand, the decrease of the diameter becomes reasonable. The changes of the diameter from the single-step model during the evaporation of water can be attributed to a change of the contrast-inducing domain. At the start, the water-rich domain is contributing dominantly to the contrast, while at the end of stage I the contrast between RF (with partially embedded PEO) and PPO is responsible for the lower diameter. This is in accordance with a roughened interface marking the transition region.

Stage II. The ceasing of the drying process at ∼150°C is indicated by 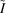 _Bragg_ becoming constant and remaining unchanged during Stage II. Furthermore, 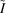 _diff_ shows a slight linear increase, which we attributed to thermally induced defects such as micropores within the matrix and perhaps also in the (still filled) pore space. The lattice parameter *a* increases slightly during stage II, which we attribute mostly to thermal expansion of the whole mesostructure during temperature increase. It has also been reported that swelling of polymers in N_2_ atmosphere might occur (Weber *et al.*, 2010 ▸, 2008 ▸; Morak *et al.*, 2017 ▸), which may be an alternative (or concomitant) effect explaining the slight increase in the lattice parameter. The power-law exponent α also remains constant during stage II, indicating no significant changes at the interface between the now fully dried micelles within the RF matrix. We conclude that, in the absence of O_2_, no surfactant removal or other major structural changes occur during stage II.

Stage III. The integrated intensity 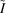 _Bragg_ does not increase immediately after O_2_ injection, but only after a delay time, which is longer the lower the temperature (see Fig. 3 ▸, and Figs. S3 and S4). In contrast to the integrated Bragg intensity, the lattice parameter and α exhibit a sudden change in slope right after oxygen is provided to the system. It is also somewhat unexpected that the diffuse intensity continues rising at a similar rate as in stage II, despite the isothermal condition in stage III. During heating in stage II, the increase in 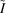 _diff_ was attributed to thermally introduced defects. We speculate that in stage III a different more subtle mechanism might be at play, since also no surfactant removal takes place, as indicated by the still constant 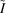 _Bragg_. We suggest that, at the beginning of oxidation, O_2_ induces complex structural changes in the interface region leading to an apparent roughening, an overall shrinkage and an increase in diffuse scattering, yet no noticeable amount of surfactant is removed.

Stage IV. We relate this stage to the actual calcination process, *i.e.* the removal of the triblock-copolymer micelles and associated creation of mesopore space. This significantly increases the SAXS contrast, as we now have 2D hexagonally arranged mesopores within the remaining RF frame. With increasing electron-density difference between the gradually emptying mesopore space and the surrounding RF matrix, the form factor and therefore the pore diameter *D* could be evaluated reliably again. As evident from Fig. 3 ▸(*a*), the lattice parameter *a* and the pore diameter *D* decrease at the same rate in stage IV (and also in stage V), which proves that during surfactant removal the material shows uniform shrinkage. The interfacial parameter α changes gradually towards −4 again but levels off at around −3.4. Even though α does not return to an ideally sharp interface corresponding to Porod’s law, this trend in stage IV indicates that the surface flattens out and becomes less corrugated as surfactants and small molecule fragments are removed.

Stage V. The plateau value of 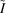 _Bragg_ suggests the completion of surfactant removal. Interestingly, it is lower than the initial value at the beginning of stage I. In terms of scattering contrast, we would expect a rather higher value, since we now have ordered mesopores within a dry RF matrix, compared with the initial scenario of the contrast of a water-rich phase versus a water-depleted polymer phase. We interpret this effect mainly as an increase in disorder of the pore structure due to the thermal treatment, which is consistent with the strong increase of the diffuse scattering, particularly in stages IV and V. The further increase of the diffuse scattering in stage V, where the Bragg scattering levels off, indicates further processes going on even after complete surfactant removal, which will, however, not be discussed or interpreted further in this work.

These considerations are not only applicable to the sample shown in Fig. 3 ▸ but also to all other samples, except the one experiencing the highest temperature of 340°C (Fig. S2). In that sample, already in stage II a non-monotonic behavior of the lattice parameter is observed. Moreover, no stage III could be identified, probably because the processes now occur too fast. Still, a plateau in the integrated Bragg intensity was observed, which is even higher compared with samples treated at lower temperatures (see Fig. 4 ▸). In general, changes of this quantity can be related to either changes in the scattering contrast between the matrix and the micelle/pore space or changes in the structural order (*e.g.* via a static Debye–Waller factor). This second case would imply an even better conservation of structural order of the mesopores in the sample subjected to the highest temperature compared with treatments at lower temperature. However, the higher plateau value of the integrated Bragg intensity for the 340°C sample could also be due to a densification of the RF matrix with temperature, which would lead to a higher electron density with respect to the (empty) pores. This is in agreement with the considerably lower lattice parameter present for this sample [Fig. 4 ▸(*b*)]. Indeed, an increase of the RF density by ∼5–10% would imply an increase of the integrated intensity by ∼10–20%, which is about the effect seen. Anyway, as the increase of the integrated intensity and therefore the thermal degradation of PEO–PPO–PEO happens within a very short time scale of 3–5 min in this sample, we regard the controllability of this set of parameters as rather limited. We therefore conclude that for a low oxygen content of 2 mol% of O_2_, a temperature interval of 260–300°C is well suited for calcination, whereas temperatures exceeding 300°C are not recommended.

Furthermore, we note that not only the final holding temperature but also the heating rate can have a noticeable influence on the final structure (see Fig. 4 ▸). However, with the current limited dataset, it was not possible to quantify the influence of the heating rate on the final structure. A systematic study of this parameter, similar to that for the temperature, is beyond the scope of this work. Future *in situ* SAXS experiments may also include *in situ* mass spectrometry to analyze the gases emitted during calcination in order to confirm the species that are released at each stage of the treatment.

Next, we discuss the influence of the oxygen content. In addition to the samples subjected to 2 mol% of O_2_ (brown colors), Fig. 4 ▸(*a*) depicts a sample subjected to 10 mol% of O_2_ (blue triangular symbols) after reaching a calcination temperature of 275°C. The increase of 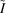 ^rel^_Bragg_ for this sample almost precisely follows that for the sample subjected to 2 mol% of O_2_ at 295°C. This leads to the conclusion that a higher oxygen content accelerates the calcination at a given temperature, as would be expected. There is, however, an important difference between the effects of temperature and oxygen content. Fig. 4 ▸(*a*) shows that there is a noticeably lower plateau value of 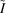 ^rel^_Bragg_ for the sample subjected to 10 mol% of O_2_, and even a slight decrease is observed for longer times. The corresponding lattice parameters [Fig. 4 ▸(*b*)], however, behave quite similarly. Several different explanations are possible to understand this effect. In terms of contrast, this could suggest a non-complete calcination with some residues of the surfactants still in the pore space, reducing the contrast with respect to the RF matrix. However, this scenario appears rather unlikely since a higher oxygen content should lead to a faster and stronger removal of the surfactants, as already rationalized earlier. The second possibility is a reduction in the electron density of the RF matrix compared with the samples with low oxygen content. This would indicate that, besides the complete removal of the micelles, part of the RF matrix is thermally decomposed, reducing its average electron density. A third possibility, probably going hand in hand with the second one, is a stronger distortion of the mesopore structure due to an overly aggressive oxygen attack, leading to a loss of structural order. Both scenarios would be detrimental, and it is therefore concluded that a high oxygen content of 10 mol% tends to increase the rate of thermal degradation of the surfactants, but at the same time also influences the delicate RF frame during the calcination stages III and IV. The almost complete degradation of the entire ordered structure (schematically depicted in Fig. 5 ▸ as stage VI) was observed for a sample treated at a high temperature of 335°C under a high oxygen content of 10 mol% of O_2_ (see Fig. S6). In this case, after a very fast calcination after ∼100 min, the Bragg intensity quickly decreases towards zero, which means that the ordered mesopore structure is destroyed. However, the comparability of this sample with other samples studied in this work is limited, since oxygen was provided for the sample shown in Fig. S6 during the whole treatment, *i.e.* also during the heating stage.

## 5. Conclusions

The aim of this work was to employ *in situ* SAXS to study the influence of temperature and the ratio of N_2_/O_2_ atmosphere on the calcination behavior of a Pluronic F127/resorcinol–formaldehyde direct soft-templated carbon precursor material. Five characteristic calcination stages were identified and the dominant mechanisms during each stage were discussed regarding their influence on the final calcined mesostructure. When the material was heated in N_2_ atmosphere, with O_2_ addition only after reaching the final calcination temperature, it was found that:

(i) An oxygen addition of 10 mol% upon reaching the final calcination temperature leads to fast decomposition and distortions of the structural integrity of the matrix during surfactant removal already for a quite low temperature of 275°C.

(ii) For an oxygen content of 2 mol%, calcination temperatures between 260 and ∼300°C influence the calcination time, but do not affect the gentle and safe surfactant removal.

The ‘simple’ experimental protocol of heating under inert atmosphere and then performing calcination under isothermal conditions was employed in order to better understand the basic mechanisms and structural changes during the different stages. Whether similar effects occur when the heating (including initial dehydration) takes place in an atmosphere already containing oxygen needs to be further investigated.

## Supplementary Material

Supporting information. DOI: 10.1107/S1600576723003886/uu5006sup1.pdf
