# Supplementary material for: Optimizing surfactant removal from a soft-templated ordered mesoporous carbon precursor: an in situ SAXS study
Source: J Appl Crystallogr. 2023 May 29;56(Pt 3):801–9. doi: 10.1107/S1600576723003886 (PMC10241053; doi:10.1107/S1600576723003886)
Supplement: Supplementary file 1 [file j-56-00801-sup1.pdf]

JOURNAL OF APPLIED CRYSTALLOGRAPHY

Volume 56 (2023)

Supporting information for article:

Optimizing surfactant removal from a soft-templated ordered mesoporous carbon precursor: an *in situ* SAXS study

Max Valentin Rauscher, Malina Seyffertitz, Richard Kohns, Sebastian Stock, Heinz Amenitsch, Nicola Huesing and Oskar Paris

S1. Data treatment

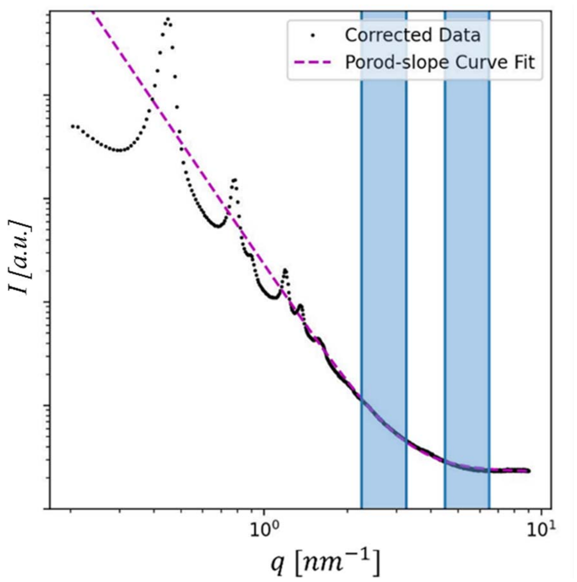

Figure S1 Example power law fit using equation 7 in the highlighted blue area, as described in the SAXS data treatment and fitting procedure.

Figure S1 shows the area where equation 7 was evaluated and depicts the fitted curve. The part between the two blue regions was excluded because of some remnants of a non-perfectly subtracted peak from the Kapton window of the SAXS flight tube. Values above q = 6.5 nm^{-1} were omitted because of a starting increase of the SAXS curve, presumably due to an intermolecular structure factor of the material.

S2. Structural parameters and integrated intensities

Figures S2-S5 depict the full evaluation of the in-situ SAXS data for the four samples presented in Figure 4. The mesostructure/mesopore diameter is not shown.

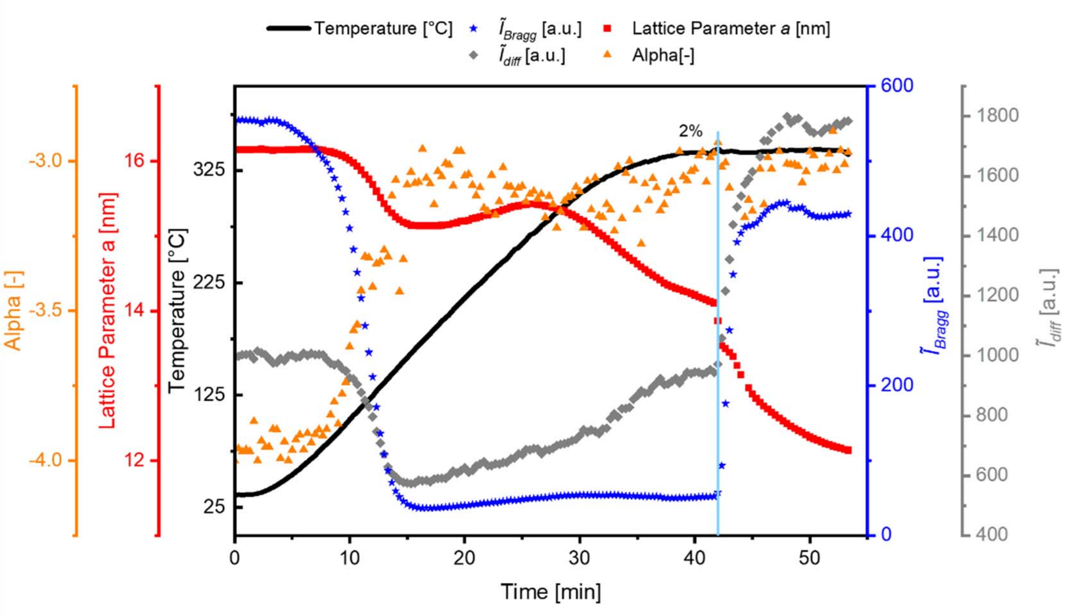

Figure S2 Progression of in-situ SAXS derived structural parameters and integrated intensities for heating in N_2 atmosphere followed by calcination at 340 °C in a 2 mol-% O_2 and N_2 atmosphere.

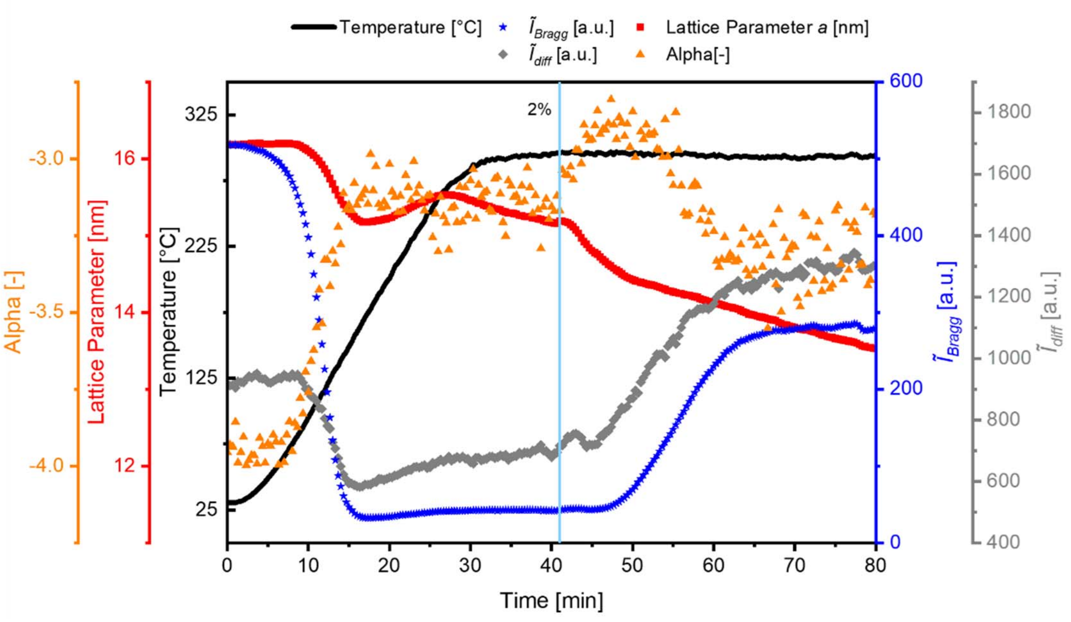

Figure S3 Progression of in-situ SAXS derived structural parameters and integrated intensities for heating in N_2 atmosphere followed by calcination at 290 °C in a 2 mol-% O_2 and N_2 atmosphere.

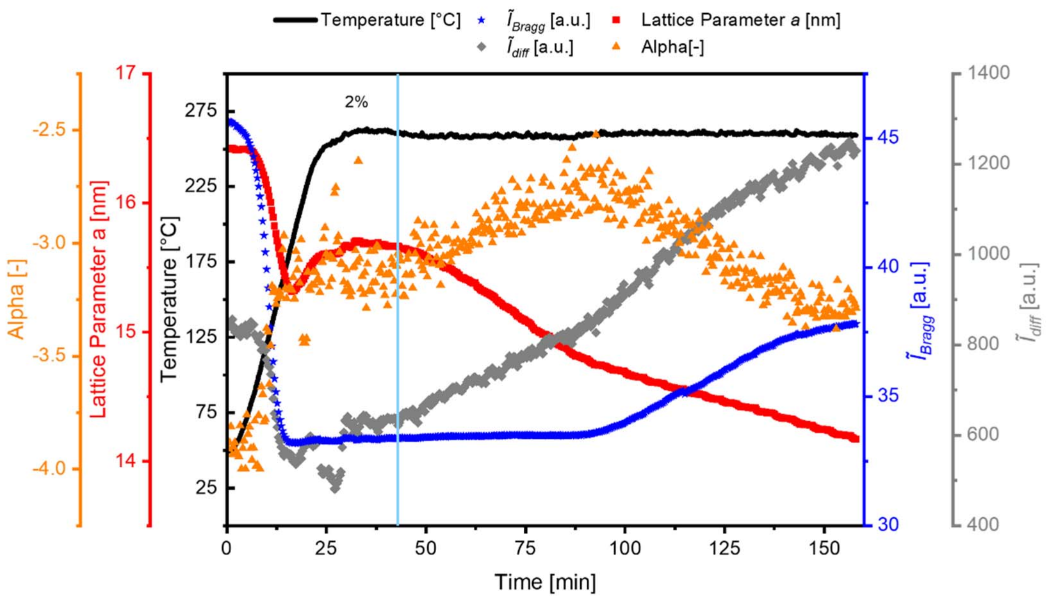

Figure S4 Progression of in-situ SAXS derived structural parameters and integrated intensities for heating in N_2 atmosphere followed by calcination at 260 °C in a 2 mol-% O_2 and N_2 atmosphere.

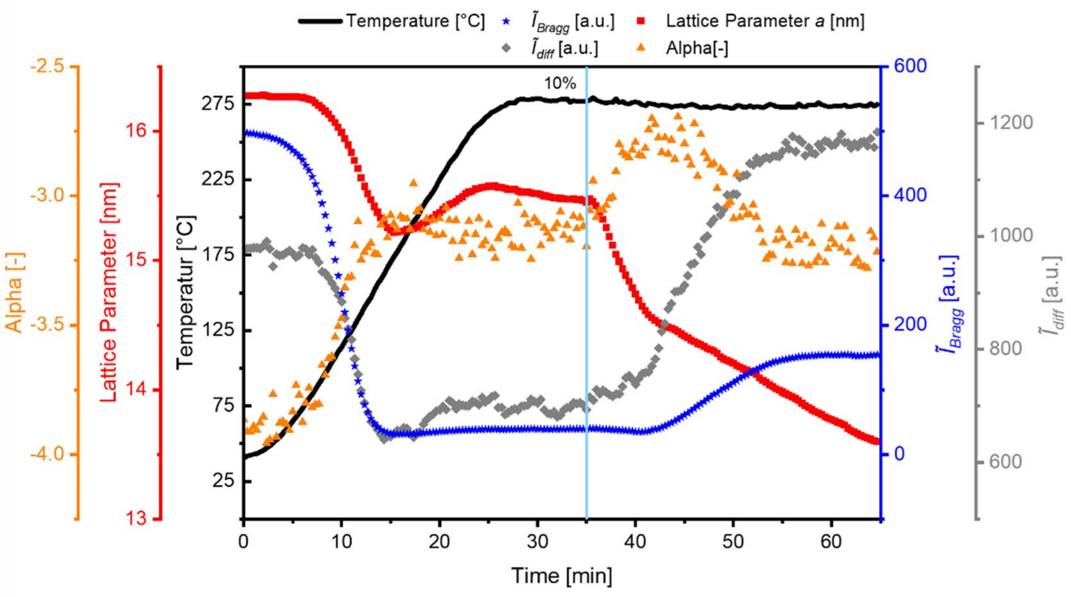

Figure S5 Progression of in-situ SAXS derived structural parameters and integrated intensities for heating in N_2 atmosphere followed by calcination at 275 °C in a 10 mol-% O_2 and N_2 atmosphere.

S3. Structural degradation

Figure S6 shows the evaluation of the in-situ SAXS parameters for a sample heated at high temperature under a high oxygen content. Please not that differently from all other samples, here the

oxygen was provided from the very beginning of heating and not only after reaching the final temperature.

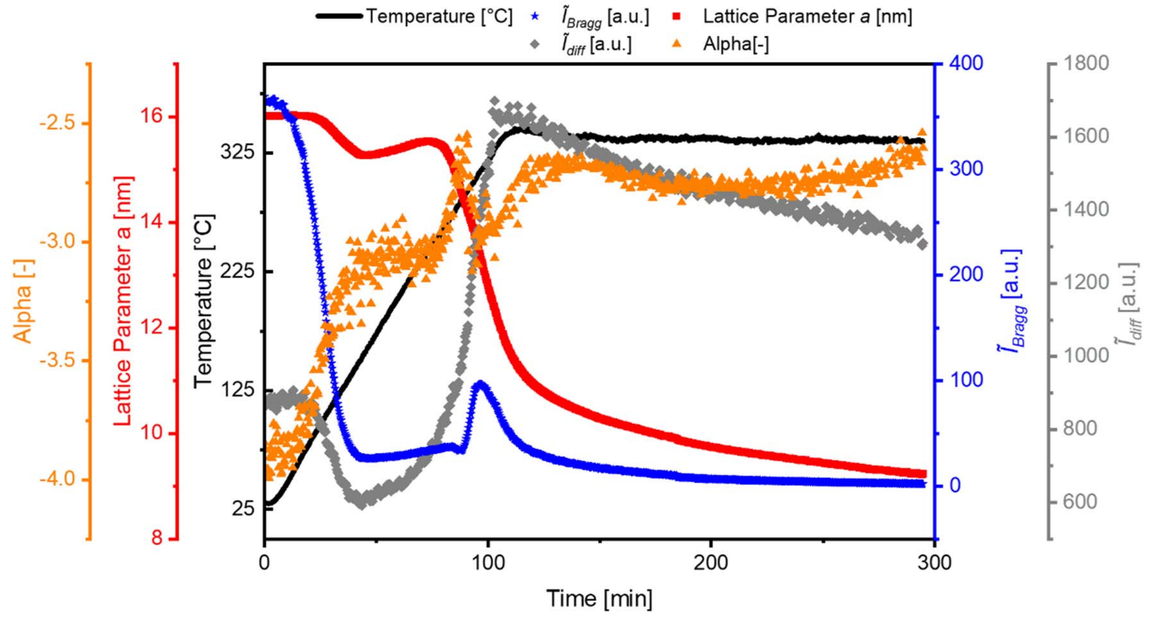

Figure S6 Progression of in-situ SAXS derived structural parameters and integrated intensities for heating in a 10 mol-% O_2 and N_2 atmosphere to a temperature of 335 °C.
